# Does Parental Investment Shape Adult Children’s Fertility Intentions? Findings From a German Family Panel

**DOI:** 10.3389/fsoc.2021.693119

**Published:** 2021-06-18

**Authors:** Antti O. Tanskanen, Mirkka Danielsbacka

**Affiliations:** ^1^ Department of Social Research, University of Turku, Turku, Finland; ^2^ Population Research Institute, Helsinki, Finland

**Keywords:** adult children, fertility intentions, fixed-effect regression, pairfam, parental investment

## Abstract

Parents can play an important role in the childbearing plans of adult children. However, studies testing whether changes in parental investment are associated with subsequent changes in fertility intentions over time are lacking. We investigated whether parental investment, measured as contact frequency, emotional closeness, financial support, and childcare, is associated with adult children’s intentions to have a first and a second, or subsequent, child within the next 2 years. These associations were studied in four different parent-adult child dyads based on the sex of parents and adult children (i.e, mother-daughter, mother-son, father-daughter, father-son). The participants are from the German Family Panel, which is a longitudinal survey of younger and middle-aged adults with eight follow-up waves. We exploited within-person (or fixed-effect) regression models, which concentrated an individual’s variation over time (i.e., whether changes in parental investment frequencies are associated with subsequent changes in adult children’s fertility intentions). It was detected that increased emotional closeness between fathers and daughters was associated with increased adult daughter’s intentions to have a first child but father-daughter contact decreased daughter’s intentions to have another child, and maternal financial support decreased son’s intentions to have a first child. Overall, statistically nonsignificant associations outweighed significant ones. Although it is often assumed that parental investment is an important factor influencing the childbearing decisions of adult children, the present findings indicate that parental investment may not increase adult children’s intentions to have a/another child in Germany.

## Introduction

In recent decades, fertility rates have declined globally, and in many countries, they are currently below the replacement level of 2.1 children per woman (e.g., Balbo et al., 2013). For instance, in Germany fertility rates have varied between 1.3 and 1.6 children per woman during the first 2 decades of the 21st century (OECD, 2021). In the current era of declining fertility, factors potentially influencing childbearing decisions have received increasing attention in different disciplines and it is often considered that receiving support from own parents could be a key factor influencing childbearing intentions on adult children. The present study uses longitudinal data from Germany to investigate the association between parental investment and fertility intentions of adult children.

Social scientists have suggested that the reason why parental support should improve younger adult’s plans to have children is the fact that this support will decrease the childbearing costs by helping individuals to alleviate conflicts between childbearing and paid work or tertiary education (e.g., Del Boca, 2002). Moreover, it has been argued that parental investment can compensate for the lack of publicly provided support, meaning that parental investment may boost childbearing especially in countries with low levels of public support (e.g., Aassve et al., 2012; Thomese and Liefbroer, 2013). That said, however, a study from Germany detected that access to public child care was not associated with improved childbearing intentions but informal support from parents was (Hank and Kreyenfeld, 2003). This indicates that parental investment may play a crucial role in childbearing decisions of adult children also in socio-economic context like Germany, where relatively generous public support for families exist (Saraceno, 2011; Saraceno, 2018).

Evolutionary scholars have argued that parental investment can influence fertility decisions of adult children in both traditional and contemporary societies (e.g., Sear and Coall, 2011; Tanskanen and Danielsbacka 2019). In the broad sense, parental investment can be defined as any investment parents channel towards their offspring (Rotkirch, 2018; see Trivers, 1972 for the original definition of parental investment). Parental investment is indicated here by financial support and childcare help parents give to their adult children as well as contact frequency and emotional closeness between parents and adult children. According to inclusive fitness theory (Hamilton, 1964) individuals can increase their own fitness (i.e., number of descendants) by not only reproducing themselves but also supporting the reproductive efforts of close kin. In this sense parents should have great interest to invest in their offspring and support the childbearing of their adult children.

In a present-day affluent societies parents still need support to raise children. However, relatives are not necessarily any longer the only or even the most important “alloparents” as several other supporters, for instance, friends, neighbors and formal childcare providers can step forward when needed (Emmott and Page, 2021; Perry and Daly, 2017). Although the number of potential helpers has diversified and widened substantially in content, the evolved psychology of humans could still be very sensitive to specific cues that have been crucial in our evolutionary past. Evolutionary scholars have argued that parental investment could play a key role in adult children’s childbearing decisions because the availability of parental investment may signal to adult children that having children is a potential option to consider (e.g., Schaffnit and Sear, 2017) and, because of the evolved tendencies of humans, pro-natal signals received from kin can effectively influence to childbearing decisions of younger adults (Newson et al., 2005; Newson et al., 2007).

Although fertility intentions are not always realized (e.g., Harknett and Hartnett, 2014), prior studies indicate that parity progression intentions (i.e., whether participants intent to have a/another child) tend to provide a fairly good proxy for actual childbearing (Balbo et al., 2013). In particular, parity progression intentions can be reliable predictors for actual childbearing when a specific time frame (e.g., 2 years) is specified (Billari et al., 2009; Philipov, 2009). Finally, although individuals who have the strongest intentions to have a child in the near future also most likely experience a family addition, the negative fertility intentions predict the actual childbearing even better, meaning that those who have negative childbearing plans rarely experience a family addition in the near future (Kuhnt and Trappe, 2016).

Many prior studies have indicated that receiving support from kin boost intentions to have a/nother child (e.g., Lehrer and Kawasaki, 1985; Miller, 1992; Raymo et al., 2010; Balbo and Mills, 2011; Fiori, 2011; Modena and Sabatini, 2011). These studies have also indicated that not only direct support (e.g., financial or practical help) received from kin but also emotional closeness between family members may have impact fertility decisions. Moreover, some have detected that the association between parental investment and fertility decisions of adult children depend on the current parity (i.e., how many current children an individual has), although the studies have provided mixed results regarding whether parental investment boosts fertility more in higher than lower parity circumstances (e.g. Tanskanen et al., 2014). Finally, it has been detected that the potential effect of parental investment on the fertility intentions of adult children may vary by the sex of parents (i.e., mother or father) as well as the sex of children (i.e., daughter or son) (e.g., Tanskanen and Rotkirch, 2014).

Here, we study first time whether changes in parental investment frequencies are associated with subsequent changes in adult children’s fertility intentions over time. We utilize within-person regression models, which concentrate on an individual’s variation over time (Jokela et al., 2018). Using the within-person approach, we test whether it is possible to provide evidence for the prediction that an increase in parental investment causally increases fertility intentions of adult children using eight waves from a longitudinal cohort study including younger and middle-aged adults from Germany. As prior studies have indicated that associations between parental support and adult children’s fertility intentions may vary by sex, lineage, and parity, we conduct separate models for four different parent-adult child dyads (i.e., mother-daughter, mother-son, father-daughter, father son), for those who intend to enter into parenthood and those who intend to have another child.

## Methods

### Data

To detect associations between parental investment and the fertility intentions of adult children, we used data from the German Family Panel (pairfam), which offers longitudinal information on intergenerational relations, childbearing intentions, and several socioecological factors. Pairfam provided data on three birth cohorts of those who were born in 1991–1993, 1981–1983 and 1971–1973. The first pairfam wave was conducted in 2008–2009, when the cohort members were aged approximately 15–17, 25–27 and 35–37, respectively. Further data collections have been conducted annually (see Brüderl et al., 2016; Huinink et al., 2011 for full data description). In the pairfam wave 2, the panel attrition was 23%, and in subsequent waves, it stabilized to approximately 10%, which is a normal attrition rate when compared to other German panel studies (Müller and Castiglioni, 2015). The achieved pairfam samples varied between 12,402 respondents in the first wave and 4,727 respondents in the eighth wave.

### Sample

We have excluded the youngest generation from the analytic sample, as they are so young that they had not yet considered starting a family in the first few data waves, and thus, retaining them may bias the results. In addition, only heterosexual respondents with a partner were included. Individuals who were pregnant or whose partners were pregnant during the survey collection, as well as those who stated that they or their partners are infertile, were excluded. We included all person-observations from respondents who have data available concerning all the studied variables. After these selections, the data of 16,400 person-observations from 4,500 unique people remained.

### Measures

Our dependent variable indicated respondent’s fertility intentions (see, e.g., Kuhnt et al., 2021; Wagner et al., 2019; Vidal et al., 2017 who have also studied fertility intentions with pairfam data). In the pairfam questionnaires, respondents were asked the following: “Do you intend to become a mother or father (again) over the next 2 years?.” The response categories were “yes, definitely”, “yes, maybe”, “no, rather not” and “no, certainly not,” but for the analysis, we have classified these responses into two categories: 0 = no (“no, rather not” and “no, certainly not”) and 1 = yes (“yes, definitely” and “yes, maybe”). The fertility intention question indicated parity progression intentions and referred to a specific period (i.e., 2 years). Thus, based on prior evidence, one can consider that these intentions could be also quite reliable predictors for actual childbearing (Billari et al., 2009; Philipov, 2009).

The main independent variables are parental investment indicators. Contact frequency and emotional closeness were measured in all eight pairfam waves and financial support and childcare in waves 2, 4, 6 and 8. All these questions were asked so that they concerned the responding person’s mothers and fathers, respectively. Contact frequency was measured through a single question by asking the respondents how often they are in contact with their parents (ranging from 0 = yearly or less often to 5 = daily). Respondents were asked to consider all types of contacts, including visits, letters, phone calls, and other types of contacts. Emotional closeness was measured using three indicators: how close the respondents felt towards their parents currently (ranging from 0 = not at all close to 4 = very close), how often the respondents told their parents what they are thinking (ranging from 0 = never to 4 = always), and how often they shared secrets or private feelings with parents (ranging from 0 = never to 4 = always) (Cronbach’s alpha for mothers = 0.81 and for fathers = 0.78). Financial support was indicated by asking how often participants receive gifts of money or valuables from parents (ranging from 0 = never to 4 = very often). Finally, it was asked how often respondents receive help with childcare from parents (ranging from 0 = never to 4 = very often): this question was asked only to those participants who had children under 15 years of age and who lived in the same household as them.

We conducted separate analyses for the four different adult child-parent sex constellations: daughter-mother, daughter-father, son-mother, and son-father. As associations between parental investment and fertility may vary by parity, we ran separate analyses for those who intended to have a first child and for those who intended to have a second or subsequent child. To obtain more robust results, we controlled for several potentially confounding variables. These covariates included the respondent’s age at interview, ethnicity, education, partner’s age at interview, partner’s education, relation duration between respondents and partners, household income quintiles, and travel time distance (in minutes) to parents. Further, we controlled for whether respondents live in East or West Germany because individuals living in these two regions tend to differ highly from each other when it comes to fertility related issues (Kreyenfeld et al., 2012). Moreover, in the analyses considering intentions to have a second or subsequent child, we controlled for respondent’s number of children and the age of the youngest child. Covariates whose values may change between study waves were modelled as time-varying variables in the within-person models. The descriptive statistics are presented in Table 1.

**TABLE 1:** Descriptive statistics over waves 1-8 in the pairfam.

	Total no	No. of persons	%	Mean (SD)	Within SD
Gender					
Male	7,251	2,021	44.2	—	—
Female	9,149	2,479	55.8	—	—
Age at interview	16,400	4,500	—	34.8 (5.40)	1.65
Ethnicity					
German native	13,244	3,575	80.8	—	—
Other	3,156	925	19.2	—	—
Education					
Lower level education	10,100	2,988	61.6	—	—
Higher level education	6,300	1,666	38.4	—	—
Area					
West Germany	10,941	3,080	66.7	—	—
East Germany	5,459	1,493	33.3	—	—
Partner age	16,400	4,500	—	35.5 (7.18)	1.85
Partner education					
Lower level education	10,352	3,119	63.1	—	—
Higher level education	6,048	1,670	36.9	—	—
Relationship duration (in months)	16,400	4,500	—	122.1 (80.51)	21.70
Family income deciles	16,400	4,500	—	6.2 (2.66)	1.16
Number of children^a^	11,586	3,218	—	1.8 (0.80)	0.23
Age of youngest child^a^	11,586	3,218	—	5.7 (4.47)	1.56
Travel time distance mother					
Living in the same house	1,861	729	11.4	—	—
Less than 10 min	4,502	1,554	27.5	—	—
10–30 min	3,714	1,447	22.7	—	—
30–60 min	1,883	780	11.5	—	—
1–3 h	1,993	688	12.2	—	—
3 h or more	2,447	809	14.9	—	—
Contact mother	16,400	4,500	—	4.6 (1.15)	0.51
Emotional closeness mother	16,400	4,500	—	2.18 (0.95)	0.47
Financial support mother^a^	9,176	4,151	—	0.59 (0.97)	0.54
Childcare mother^a^ ^,^ ^b^	6,208	2,891	—	1.81 (1.28)	0.56
Travel time distance father					
Living in the same house	1,399	566	10.3	—	—
Less than 10 min	3,656	1,257	26.8	—	—
10–30 min	2,943	1,196	21.6	—	—
30–60 min	1,592	668	11.7	—	—
1–3 h	1,824	621	13.4	—	—
3 h or more	2,219	765	16.3	—	—
Contact father	13,649	3,837	—	4.3 (1.30)	0.56
Emotional closeness father	13,649	3,837	—	1.78 (0.92)	0.47
Financial support father^a^	7,619	3,513	—	0.57 (0.96)	0.55
Childcare father^a^ ^,^ ^b^	5,097	2,417	—	1.42 (1.25)	0.53

Notes. Total no. = Number of total person-observations; No. of persons = Number of unique persons.

SD = Overall standard deviation; Within-person SD = Within-person standard deviation.

a = Waves 2, 4, 6 and 8.

b = Only participants with <15-year-old children are included.

We analyzed the longitudinal pairfam data by using multilevel linear regression models in which the repeated measures (i.e., person-observations) are nested within the respondents. Although our dependent variable was dichotomous, we did not use logit models due to their limitations (see Mood, 2010 for discussion). However, sensitivity analyses conducted with logistic regressions provided similar results as with linear regressions. We ran within-person (or fixed-effect) regression models, which show the individual’s variation over time (Jokela et al., 2018). This study primarily aimed to investigate whether changes in parental investment frequencies are associated with subsequent changes in adult children’s fertility intentions. To study this question, we used within-person regression models. In within-person models, the observed participants served as their own controls, and these models eliminate all the time-invariant components (Allison, 2009), such as numerous genetic factors and other selection effects. Thus, within-person models provide a test for causality in the associations between parental investment and adult children’s fertility intentions.

## Results

First, we provided descriptive results of the participants who had within-person data and were thus included in the fixed effect models (i.e., participants who reported changes in parental involvement between study waves). According to the transition probabilities of different parental involvement factors, individuals often remained in the same category, and when changes occurred, there was more often a transition between categories close to each other than those further apart (results not shown in tables and available from corresponding author). The stability and change in fertility intentions was measured by intraclass correlations that showed the correlation of the person-observations within a person over time. The intraclass correlations in fertility intentions varied between 0.63 and 0.76, which indicated relatively strong stability between the study waves.

Then we proceed to the results from the within-person regression models. We examined first whether parental investment is associated with adult daughter’s intentions to have a/another child and the results are presented in Table 2. We were unable to find significant associations between mother’s investment and daughter’s intentions to have a/nother child. This was it in the case of all investment variables used (i.e., contact frequency, emotional closeness, financial support, and childcare). When it comes to father-daughter relations, Table 2 showed that increased emotional closeness between fathers and daughters was associated with daughter’s increased intentions to have a first child. However, increased contact frequency between fathers and daughters was associated with daughter’s decreased intentions to have a second or subsequent child. We were unable to find any other significant association in other models.

**TABLE 2:** Parental investment and adult daughters' intentions to have a/another child over waves 1-8 in the pairfam.

First child	Maternal investment			Paternal investment		
	β	95% CI		β	95% CI	
		Lower	Upper		Lower	Upper
Contact	−0.01	−0.05	0.02	0.001	−0.03	0.03
Emotional closeness	0.02	−0.02	0.05	0.04	0.01	0.08
Financial support	−0.01	−0.04	0.03	−0.03	−0.07	0.01

Second or subsequent child	Maternal investment			Paternal investment		
	β	95% CI		β	95% CI	
		Lower	Upper		Lower	Upper
Contact	−0.01	−0.02	0.01	−0.01	−0.02	−0.001
Emotional closeness	−0.01	−0.02	0.003	−0.0004	−0.01	0.01
Financial support	−0.01	−0.03	0.01	−0.01	−0.03	0.01
Childcare	0.01	−0.003	0.03	0.001	−0.02	0.02

Notes. Values are β-coefficients (and 95% confidence intervals) of within-person regressions. Mother-daughter contact and emotional closeness: First child *n* = 2,221 person-observations from 749 persons; Second or subsequent child *n* = 6,858 person-observations from 1,898 persons. Maternal financial support: First child *n* = 1,272 person-observations from 680 persons; Second or subsequent child *n* = 3,805 person-observations from 1,726 persons. Maternal childcare: Second or subsequent child *n* = 3,612 person-observations from 1,175 persons. Father-daughter contact and emotional closeness: First child *n* = 1,935 person-observations from 659 persons; Second or subsequent child *n* = 5,610 person-observations from 1,588 persons. Paternal financial support: First child *n* = 1,102 person-observations from 594 persons; Second or subsequent child *n* = 3,116 person-observations from 1,444 persons. Paternal childcare: Second or subsequent child *n* = 2,997 person-observations from 1,405 persons.

Next, we examined associations between parental investment and adult son’s fertility intentions. Table 3 showed that increased financial support from mothers to adult sons was associated with son’s decreased intentions to have a first child. However, parental investment was not associated with adult son’s childbearing intentions in any other models. This was it whether we considered the investment of mothers or fathers and also according to parity.

**TABLE 3:** Parental investment and adult sons' intentions to have a/another child over waves 1-8 in the pairfam.

First child	Maternal investment			Paternal investment		
	β	95% CI		β	95% CI	
		Lower	Upper		Lower	Upper
Contact	0.004	−0.03	0.04	0.001	−0.03	0.03
Emotional closeness	0.02	−0.01	0.05	−0.004	−0.04	0.03
Financial support	−0.05	−0.10	−0.01	−0.03	−0.07	0.01

Second or subsequent child	Maternal investment			Paternal investment		
	β	95% CI		β	95% CI	
		Lower	Upper		Lower	Upper
Contact	0.002	−0.01	0.02	0.00004	−0.02	0.02
Emotional closeness	0.01	−0.01	0.02	0.01	−0.01	0.03
Financial support	−0.01	−0.03	0.02	−0.01	−0.03	0.02
Childcare	0.01	−0.01	0.03	0.01	−0.01	0.03

Notes. Values are β-coefficients (and 95% confidence intervals) of within-person regressions. Mother-daughter contact and emotional closeness: First child *n* = 2,592 person-observations from 929 persons; Second or subsequent child *n* = 4,439 person-observations from 1,241 persons. Maternal financial support: First child *n* = 1,471 person-observations from 840 persons; Second or subsequent child *n* = 2,464 person-observations from 1,148 persons. Maternal childcare: Second or subsequent child *n* = 2,407 person-observations from 1,131 persons. Father-daughter contact and emotional closeness: First child *n* = 2,271 person-observations from 820 persons; Second or subsequent child *n* = 3,627person-observations from 1,056 persons. Paternal financial support: First child *n* = 1,287 person-observations from 740 persons; Second or subsequent child *n* = 1,998 person-observations from 956 persons. Paternal childcare: Second or subsequent child *n* = 1,966 person-observations from 947 persons.

## Discussion

This study investigated associations between parental investment and the fertility intentions of younger and middle-aged adults from Germany. Parental investment was indicated by contact frequency, emotional closeness, financial support and childcare help, which have been commonly used variables also in prior studies on parental investment (Rotkirch, 2018). We indicated fertility intentions by a measure demonstrating participant’s intentions to have a/another child during the following 2 years; this indicator has been used also in several recent studies considering childbearing plans of younger and middle-age adults (Kuhnt et al., 2021; Wagner et al., 2019; Vidal et al., 2017). Four different parent-adult child dyads that accounted for the sex of the parents and their adult children were detected. We conducted within-person regression analyses, which provide a sophisticated way to study person-specific changes over time (Jokela et al., 2018).

It was detected that statistically nonsignificant associations tend to outweigh significant associations. Only three significant associations were present in within-person models, and two of them indicated that parental investment decreases intentions to have a/another child. The increased amount of contact between fathers and daughters was associated with daughter’s decreased intentions to have a second or subsequent child and increased maternal financial support with son’s decreased intentions to enter into parenthood. The only positive within-person association indicated that increased emotional closeness between fathers and daughters increased adult daughter’s intentions to have a first child.

One explanation for the two negative within-person effects (i.e., father-daughter contact decreased daughter’s intentions to have another child, and maternal financial support decreased son’s intentions to enter into fatherhood) could be that parental investment serves as a response to increased need for help of adult children (Szydlik, 2016). Thus, parental support may increase during the times when adult children experience an unstable phase of life, and simultaneously, intentions to have children may decrease. It is not clear, however, why similar associations were not present when parental investment was measured with other factors. Making the interpretation even more challenging, it was found that when emotional closeness between fathers and daughters increased, daughter’s intentions to have a first child also increased. Thus, it cannot be ruled out that the within-person effects found here are only chance findings.

In most cases, we were unable to detect within-person associations between parental investment and the fertility intentions of adult children, which also indicates that there may not be causal association between parental investment and adult children’s childbearing intentions. This finding was in contrast with theories predicting that such association should exist (see Tanskanen and Danielsbacka, 2019 for discussion). However, it could be that the mere presence of potential kin helpers (i.e., own mother or father is alive and able to help) could influence on fertility intentions and, thus, the changes in kin support in short term is not that crucial (Tanskanen and Rotkirch, 2014). Studies from pre-modern and historical populations have shown that parental presence in the same village is associated with increased fertility of adult children (e.g., Chapman et al., 2021). However, future studies are needed to investigate the causes for the lack of within-person effects noted in the present study.

Compared to previous studies that have detected associations between parental involvement and the fertility of adult children, the present study has several strengths. We have analyzed large-scale and population-based longitudinal data, which have gathered repeated information on the same respondents annually. The data were analyzed using fixed-effect regressions that focused on within-person variation over time and provided a test for causality in the association between parental investment and fertility intentions of adult children. In the within-person models the individuals served as their own controls and thus all time-invariant components were eliminated in the models. In addition, we were able to control for several time-variant factors. To the best of our knowledge, the within-person approach has not been used previously to analyze the association between parental investment and childbearing intentions of adult children.

Obviously, the present study is not without limitations. For instance, there could be some time-variant unobserved factors that influence parental involvement or fertility intentions that were not possible to take into account. Although we took several time-varying factors into account, all such factors are difficult, if not impossible, to control. It could also be that some changes in the parental investment or fertility intentions reported across study waves were based on either response or coding errors, meaning that changes could be either under- or overrepresented. Moreover, the surveys following the same participants over time tend to suffer from panel attrition, which may also influence the results. Regarding fertility related issues, a Swiss study found that the arrival of a child was associated with the increased dropping out of participants, which was likely related to the fact that parents of newborn children often experience heavy time constraints making them unavailable for surveys (Voorpostel and Lipps, 2011). Childbearing intentions, however, could be better fertility indicator in panel studies than actual childbearing because it may not be similarly associated with panel drop out. This is based on the fact that the intention to have a child does not increase time constraints like with actual childbearing. Indeed, investigation based on an Austrian panel study found that fertility intentions were not associated with panel drop out (Buber-Ennser, 2014).

Here, we have investigated the potential effect of parental investment on adult children’s childbearing intentions in Germany, where relatively generous public support for families exist (Saraceno, 2011; Saraceno, 2018), meaning that there is not that crucial need for parental investment compared to countries with less beneficial public support. Because of the relatively generous public support, younger and middle-aged adults in Germany are not as dependent on parental investment, meaning that their childbearing decisions could be based on other factors than opportunities to receive kin support. However, parental investment could influence fertility decisions in countries with less generous public support for families (Sear et al., 2016). Thus, there is need for comparative studies analyzing whether parental investment is associated with childbearing intentions of adult children in other countries.

Although we were unable to provide strong evidence for the prediction that parental investment increases adult children’s fertility intentions, close ties between generations may still benefit adult children in several ways. For instance, parental financial support could be a key factor making it possible for younger adults to achieve their educational plans and help them begin adult life (e.g., Hamilton, 2013; Hamilton et al., 2018). Moreover, support received from parents can substantially help to reconcile work and family life among those individuals who already have children (Arpino and Luppi, 2020). Finally, parental investment may also increase the health and wellbeing of younger adults; for instance, after the child has been born, support from older parents may help to decrease maternal depression (Hagaman et al., 2021).

To conclude, the present study provided only limited evidence for the prediction that parental investment is associated with increased childbearing intentions of adult children. We hope that our findings stimulate future studies to use longitudinal data and within-person models when investigating whether different family related variables are associated with fertility decisions.

## Data Availability

This paper uses data from the German Family Panel pairfam, coordinated by Josef Bruderl, Sonja Drobnič, Karsten Hank, Bernhard Nauck, Franz Neyer, and Sabine Walper. pairfam is funded as long-term project by the German Research Foundation (DFG). Analyses are based on data from the first eight waves of pairfam, release 8.0 (Brüderl et al., 2017). A detailed description of the study can be found in Huinink et al. (2011). This data can be found here: http://www.pairfam.de.
